# LINC01133 promotes pancreatic ductal adenocarcinoma epithelial–mesenchymal transition mediated by SPP1 through binding to Arp3

**DOI:** 10.1038/s41419-024-06876-3

**Published:** 2024-07-10

**Authors:** Yefan Yang, Yuxi Gong, Ying Ding, Shuning Sun, Rumeng Bai, Shuaishuai Zhuo, Zhihong Zhang

**Affiliations:** Department of Pathology, The First Affiliated Hospital with Nanjing Medical University, Nanjing, Jiangsu 210029 China

**Keywords:** Pancreatic cancer, Cancer microenvironment

## Abstract

Pancreatic ductal adenocarcinoma (PDAC) is a lethal disease with limited treatment methods. Long non-coding RNAs (lncRNAs) have been found involved in tumorigenic and progression. The present study revealed that LINC01133, a fewly reported lncRNA, was one of 16 hub genes that could predict PDAC patients’ prognosis. LINC01133 was over-expressed in PDAC tumors compared to adjacent pancreas and could promote PDAC proliferation and metastasis in vitro and in vivo, as well as inhibit PDAC apoptosis. LINC01133 expression positively correlated to secreted phosphoprotein 1 (SPP1) expression, leading to an enhanced epithelial-mesenchymal transition (EMT) process. LINC01133 bound with actin-related protein 3 (Arp3), the complex reduced SPP1 mRNA degradation which increased SPP1 mRNA level, ultimately leading to PDAC proliferation. This research revealed a novel mechanism of PDAC development and provided a potential prognosis indicator that may benefit PDAC patients.

## Introduction

Pancreatic cancer is a lethal disease with a five-year survival rate of less than 10% [1]. Pancreatic ductal adenocarcinoma (PDAC) is the leading histological subtype and encompasses over 90% of all Pancreatic cancers [2]. Despite the evolution of chemotherapies and targeted therapies, improvement in overall survival remains unsatisfactory. Post-operation relapse and metastasis were common in surgically treated patients and eventually led to fatality.

Recently, many studies have validated the reliability of survival prognosis models, which incorporated gene expression or mutation status. Combined with traditional prediction markers such as tumor stage and differentiation, these models have the potential for customizing targeted therapy [3, 4]. However, many of these models were constructed using bioinformatic techniques and the underlying mechanisms through which numerous key factors activate or suppress tumorigenesis remain largely uncharted.

Long non-coding RNAs (lncRNAs) represent a subset of RNA that exceeds 200 nucleotides in length, and only some lncRNAs with small open reading frames could encode functional polypeptides or proteins [5, 6]. LncRNAs can influence both transcriptional and post-transcriptional regulation networks, and dysregulated lncRNAs are involved in many human pathological processes including cancer [7]. Many lncRNAs have been found dysregulated in association with the progression of PDAC through a wide array of biological processes. For instance, lncRNA MACC1-AS1 elevation has been linked with NOTCH1 signaling pathway activation, resulting in unfavorable outcomes in PDAC [8]. LncRNA PACERR polarized tissue-associated macrophages in PDAC by modulating the KLF12/p-AKT/c-myc pathway [9]. Furthermore, lncRNAs HIF1A-AS1 and PVT1 have been implicated in fostering gemcitabine resistance of PDAC [10, 11].

Positioned on the genetic map at 1q23.2, LINC01133 emerges as a noteworthy lncRNA in recent studies. It has diverse expressions and is dysregulated in various cancers. Elevated LINC01133 level was observed in lung cancer, hepatocellular carcinoma and gynecological cancers, and correlated with augmented proliferation potential [12].

The epithelial-mesenchymal transition (EMT) is a cellular process in which epithelial cells relinquish the need for neighboring cell contact, adopting mesenchymal attributes. The process is associated with tumor initiation, proliferation, and resistance to therapy [13]. LncRNAs were found capable of regulating EMT and therefore have potential as biomarkers or therapeutic targets [14]. LINC01133 was found to participate in EMT, but its mechanisms are varied, leaving room for further illumination [15–18].

In the present study, we constructed a novel PDAC gene expression model to predict prognosis. This model directed our attention towards a hub gene, LINC01133, prompting a comprehensive exploration into its role in PDAC. Later, we discovered its unique interaction with secreted phosphoprotein 1 (SPP1) which led to an enhanced EMT process in PDAC. Our study further demonstrated that LINC01133, which is bound with Arp3 and the complex regulated the downstream SPP1, could be elevated through activation of c-Jun in the Wnt/β-catenin pathway.

## Methods

### Online data collection

PDAC RNA sequencing data, clinical and survival information of TCGA dataset were downloaded from by Xena database (https://xenabrowser.net/datapages/). Additionally, two PDAC microarray datasets, GSE32676 and GSE61166, were selected and downloaded from the GEO database (http://www.ncbi.nlm.nih.gov/geo/).

### Construction of a PDAC prognosis prediction model

Differential expression analysis was carried out using the ‘limma’ package in R 4.2.2 across the 3 aforementioned datasets. Genes with 0 expression in over 50% of samples were filtered out. Samples with a follow-up duration of less than 30 days were excluded. Differentially expressed genes (DEGs) consolidated from 3 datasets were submitted to univariable Cox regression analysis and lncRNA with a *P* value of less than 0.1 and met proportional hazard assumption were chosen for stepwise multivariable Cox regression using the ‘survival’ package in R to construct a prognostic model. The performance of the model was evaluated by the receiver operating characteristic curve and area under the curve using the R package “survivalROC”. The risk score was calculated for each patient using the model, and high-risk and low-risk patients were grouped according to the median risk score.

### Tissue sample and clinical data collection

Forty pairs of PDAC tumors and adjacent normal pancreas tissue were obtained from patients underwent radical pancreatectomy without pre-operation treatment. The study was approved by the Ethics Committee of the First Affiliated Hospital of Nanjing Medical University (2017-SRFA-049). Written consent was obtained from all patients and all procedure was conducted according to the Declaration of Helsinki.

### RNA extraction and qRT-PCR

Total RNA was extracted using TRIzol reagent (TaKaRa, Shiga, Japan). Reverse transcription was performed using PrimeScript^TM^ RT reagent Kit with gDNA Eraser (TaKaRa). The TB Green^TM^ Premix Ex Taq^TM^ (TaKaRa) was used to conduct qRT-PCR. U6 small nuclear RNA was adopted as the internal control. Primers were designed and synthesized by Invitrogen (Shanghai, China). Primer sequences were listed in Supplementary Table S1.

### Western blot analysis

Total proteins were extracted from cells using lysis buffer (RIPA, Millipore, Billerica, MA, USA) and separated by sodium dodecyl sulfate-polyacrylamide gel electrophoresis (SDS-PAGE). Antibodies include Arp3, SPP1, vimentin, E-Cadherin, and N-Cadherin were purchased from Proteintech (Wuhan, China). β-actin (Beyotime, Shanghai, China) was used as an endogenous reference.

### Statistical analysis and large language model usage

R 4.1.2 was used to perform statistical analysis. Continuous variables between groups were compared using a two-sided Mann–Whitney *U* test or Student’s *t*-test depending on normal distribution status. Categorical variables between groups were compared using χ2 or Fisher’s exact test as appropriate. Kaplan–Meier method and log-rank test were used to determine the significance of survival analysis and median overall survival. *P* < 0.05 was considered statistically significant. During the preparation of this work, the authors used ChatGPT in order to improve language and readability. The authors reviewed and edited the content and took full responsibility for the content of the publication.

Details for cell culturing, transfection, functional experiments, animal model construction, RNA-sequencing, immunofluorescence and fluorescence in situ hybridization, RNA pull-down and immunoprecipitation (RIP) assay, see Supplementary Methods.

## Results

### Constructing a PDAC prognosis model with gene expression signature and model assessment

**Fig. 1:**
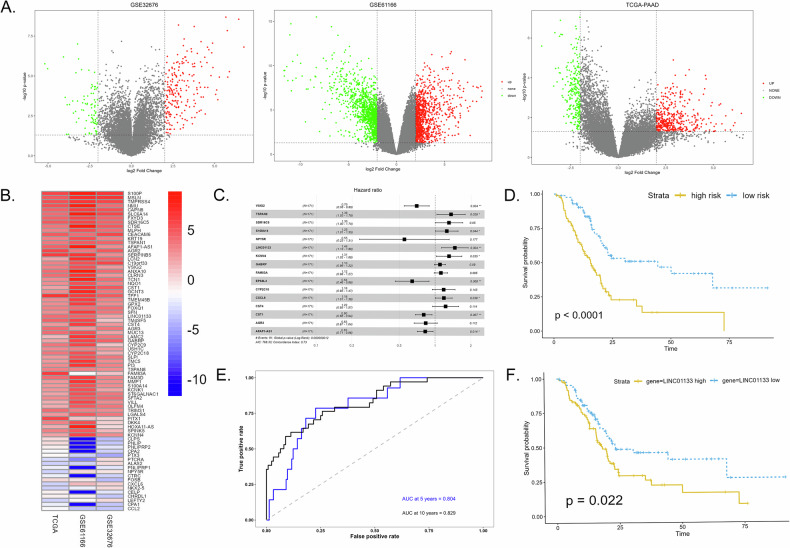
Constructing a PDAC prognosis model based on gene expression signature. **A** Volcano plot of differentially expressed genes (DEGs) in datasets GSE32676, GSE61166, and TCGA database. **B** Integrated DEGs from the 3 datasets using the robust rank aggregation method. **C** Identification of 16 genes included in the prognostic model through stepwise multivariable Cox regression. **D** The Kaplan-Meier curve illustrated the overall survival in high and low-risk groups. **E** The receiver operating characteristic (ROC) curve of the risk model. **F** The Kaplan-Meier curve of the TCGA cohort stratified according to LINC01133 expression levels.

Differential expression analysis was performed on the TCGA, GSE32676, and GSE61166 datasets using the ‘limma’ package. 625, 297, and 2 183 DEGs between PDAC samples and adjacent normal samples were identified, respectively, with a threshold of |log2 Fold Change | > 2 and *P* value < 0.05 (Fig. 1A). Sixty-four upregulated and 25 downregulated DEGs were selected after integrating the 3 datasets using the robust rank aggregation method (Fig. 1B) [19]. Eventually, 16 genes were selected out of 89 integrated DEGs by Cox regression, and a prognosis stratification model was constructed (Fig. 1C). Nine genes showed a *P* value less than 0.05 and were independent prognostic factors ().

The TCGA cohort was divided into high/low-risk groups based on the risk score generated from the prognosis model. Kaplan–Meier analysis revealed a significantly shortened survival time in the high-risk group compared to the low-risk group (Fig. 1D). The AUC of ROC at 5-year and 10-year were 0.804 and 0.829, respectively, proving the model’s robustness in prognosis prediction (Fig. 1E).

Comparing the clinicopathological features between different risk groups found patient’s age, gender, evaluated lymph nodes and positive lymph nodes did not differ between groups (Supplementary Table S2). The high-risk group contained more higher-grade tumors (*P* = 0.03).

### LINC01133 was upregulated in PDAC tumor tissue and cells

**Fig. 2:**
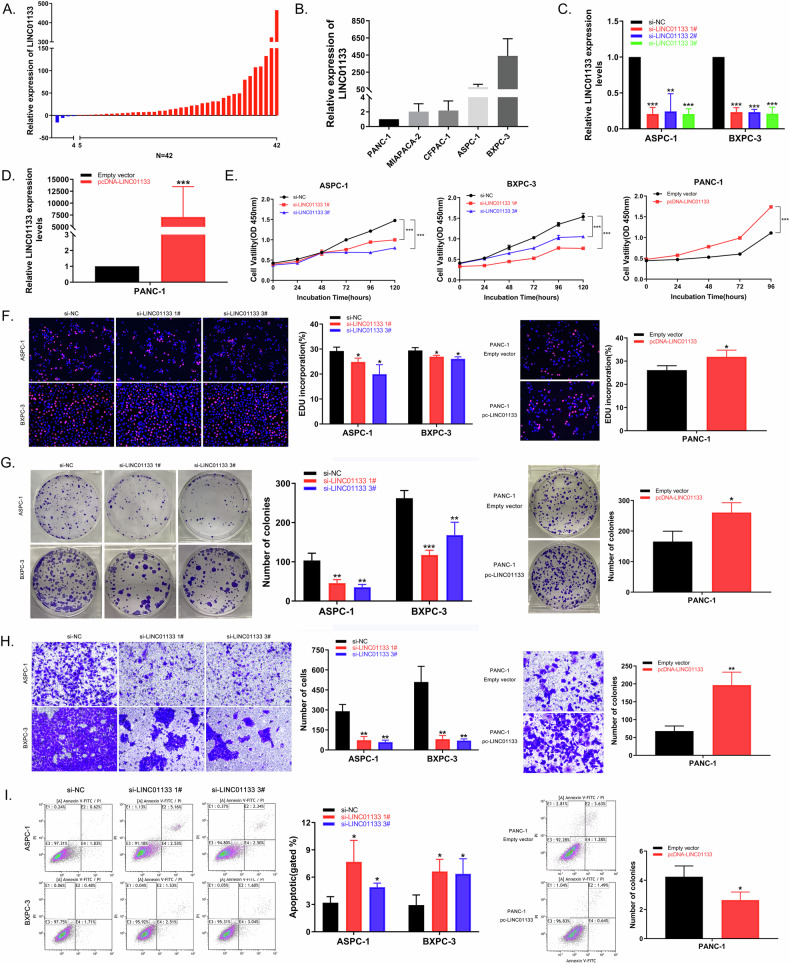
LINC01133 promoted PDAC cell proliferation, migration, and resistance to apoptosis in vitro. **A** LINC01133 expression level in PDAC tissues compared to matched adjacent normal tissue. **B** LINC01133 expression in PANC-1, MIACAPA-2, CFPAC-1, ASPC-1 and BXPC-3 cell lines. **C**-**D** Si-LINC01133 1# and 3# transfection could effectively knockdown LINC01133 expression in BXPC-3 and ASPC-1 cell lines, while pcDNA-LINC01133 transfection could over-express LINC01133 in PANC-1. **E**-**G** CCK-8, EdU, and colony formation analysis performed on BXPC-3, ASPC-1, and PANC-1 cells, transfected with si-LINC01133 and pcDNA-LINC01133 respectively, to determine cell proliferation ability. **H** Transwell analysis on BXPC-3, ASPC-1, and PANC-1 cells to determine cell migration ability after transfected with si-LINC01133 and pcDNA-LINC01133, respectively. **I** Flow cytometry analysis was used to determine the apoptosis rate of ASPC-1 and PANC-1 cells after transfected with si-LINC01133 and pcDNA-LINC01133, respectively.

Among the 16 genes included in the final model, we chose LINC01133 for further experiments, as its expression level was steadily upregulated in PDAC in three datasets. LINC01133 over-expression was an independent risk factor for PDAC and a shorter overall survival time was observed in patients with higher LINC01133 expression levels (*P* = 0.02, Fig. 1F). LINC01133 expression was validated in 42 pairs of PDAC patient samples, and LINC01133 level was higher in PDAC tissues compared to matched adjacent normal tissue (Fig. 2A). LINC01133 was highly expressed in BXPC-3 and ASPC-1, and a relatively low LINC01133 expression was observed in PANC-1, CFPAC-1, and MIACAPA-2 by qRT-PCR analysis (Fig. 2B).

### LINC01133 promoted PDAC proliferation, migration, and resistance to apoptosis in vitro

Three small interfering RNAs designed to silence LINC01133 (si-LINC01133 1#, si-LINC01133 2#, and si-LINC01133 3#) were transfected in BXPC-3 and ASPC-1 cells and transfection efficiency was verified by qRT-PCR analysis. Si-LINC01133 1# and si-LINC01133 3# displayed a more effective knockdown effect and were used for subsequent functional experiments (Fig. 2C). PANC-1 cells were transfected with LINC01133 over-expression vector (pcDNA-LINC01133), and the transfection efficiency was also confirmed by qRT-PCR analysis (Fig. 2D).

We then explored the function of LINC01133 in vitro. CCK-8 and EdU analysis revealed a decrease in PDAC cell proliferation after LINC01133 knockdown and an increase after over-expression of LINC01133 (Fig. 2E, F). Colony formation analysis showed a decrease in PDAC cell colonies after knocking down LINC01133, meanwhile, colonies increased in LINC01133 over expressed PDAC cells (Fig. 2G). An increased penetration through the basement membrane was observed in LINC01133 overexpression cells during transwell analysis, while downregulated LINC01133 significantly suppressed PDAC cell migration (Fig. 2H).

Flow cytometry analysis found LINC01133 knockdown could induce cell cycle arrest at the S phase in BXPC-3 cell line, but not in ASPC-1 and PDAC cell line (Supplementary Fig. S1). An increase in early and late apoptosis in LINC01133 knockdown cells suggested that LINC01133 may lead to PDAC cells resisting apoptosis (Fig. 2I).

### LINC01133 promoted PDAC tumor growth and metastasis in vivo

**Fig. 3:**
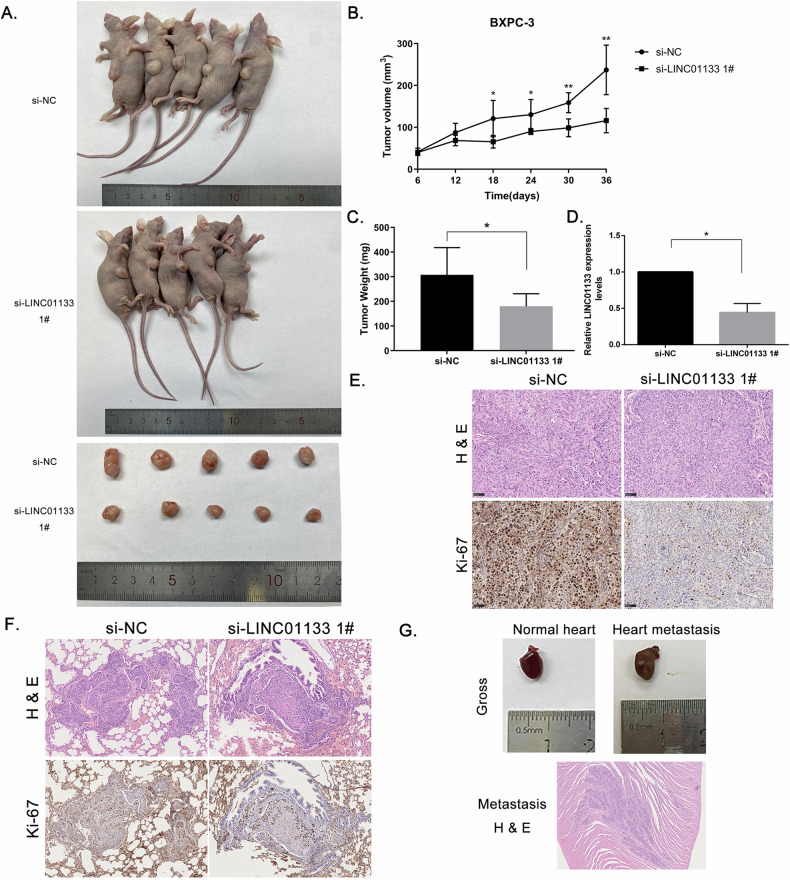
LINC01133 promoted PDAC tumor growth and metastasis in vivo. **A** Gross image of nude mice injected with BXPC-3 cells transfected with NC and si-LINC01133 1# and resected tumors. **B** Tumor volume of NC and si-LINC01133 1# group measured every 6 days. **C** Mean tumor weight and SD. **D** Relative expression of LINC01133 in xenograft tumors between 2 groups. **E** H&E staining and IHC staining of ki-67 of the xenograft tumors. Magnification, ×400. **F** Lung metastasis tumor nodule stained with H&E and IHC using ki-67 antibody. **G** Metastasis nodule in the heart of 1 mouse in NC group, grossly and microscopically. NC: negative control. IHC, immunohistochemistry. Points, mean; bars, SD. *P < 0.05, **P < 0.01.

Since LINC01133 could promote PDAC proliferation in cell lines, we thought to investigate the functional role of LINC01133 in vivo. Nude mice xenograft models were constructed using a BXPC-3 cell line. Tumor volume was measured every 2 days (Fig. 3A). Before the tumor reached 1.5 centimeters in maximum diameter, the mice were euthanized, and tumors were harvested. The si-LINC01133 1# group showed decreased tumor size and weight compared to the si-NC group (Fig. 3B, C). The qRT-PCR analysis confirmed LINC01133 expression was lower in the si-LINC01133 1# group (Fig. 3D). Immunohistochemistry staining showed a relatively lower expression of proliferation index Ki-67 in the si-LINC01133 group than in the si-NC group (Fig. 3E).

Tail vein metastasis models found metastatic tumor nodules in the lung in both groups (Fig. 3F), while the number of metastatic nodules and calculated size were higher in the NC group than in the si-LINC01133 group (42 vs 2, 1.34 mm^2^ vs 0.64 mm^2^, respectively). We also detected a metastatic lesion in the heart of a mouse in the NC group which died 12 h before the scheduled ending (Fig. 3G), while the si-LINC01133 group had no metastasis lesion in organs other than the lung. These results indicated that LINC01133 could promote tumor growth and metastasis in vivo.

### LINC01133 mediating genes were enriched in the EMT pathway and participated in the PDAC EMT process

**Fig. 4:**
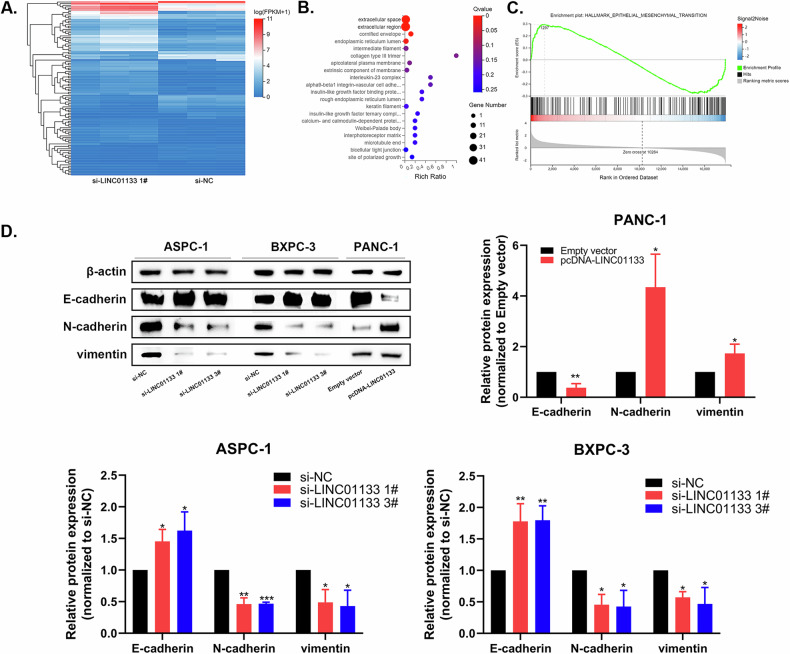
LINC01133 mediating genes were enriched in the EMT pathway and participated in the PDAC EMT process. **A** mean-centered, hierarchical clustering of RNA expression heatmap of BXPC-3 cells transfected with NC and si-LINC01133 with 3 repeats. **B** Bubble plot of enriched pathways in the cellular process using Gene Ontology analysis. **C** GSEA-hallmark enrichment plot of the EMT pathway. **D** Western blot assay and relative protein level of EMT markers E-cadherin, N-cadherin, and vimentin in LINC01133 knockdown and over-expressed groups, with control. GSEA, gene set enrichment analysis. *P < 0.05, **P < 0.01.

To determine the downstream mechanism of LINC01133 in PDAC development, transcriptome sequencing was performed on BXPC-3 cells transfected with si-LINC01133 1# and si-NC. 108 genes were differentially expressed between the control and LINC01133 knockdown group, and enrichment analysis showed that these genes were mostly enriched in extracellular space, extracellular region, cornified envelope, and endoplasmic reticulum lumen pathways (Fig. 4A, B). The gene set enrichment analysis hallmark showed that the DEGs were enriched in the epithelial-mesenchymal transition pathway (Fig. 4C). Previous studies have demonstrated that LINC01133 participated in the EMT process in colorectal cancer and PDAC tumor-derived exosomal [17, 18]. Combined with our bioinformatic analysis results, we suspected LINC01133 could promote the EMT process in PDAC.

To verify our hypothesis, western blot analysis was performed which showed an increase in the epithelial cell marker E-cadherin and a decrease in the mesenchymal cell markers N-cadherin and vimentin after LINC01133 knockdown. Subsequently, a decrease in E-cadherin and an increase in N-cadherin and vimentin was observed after over-expressing LINC01133, confirming that LINC01133 could enhance tumor EMT in PDAC (Fig. 4D).

### LINC01133 promoted tumor migration and EMT by targeting SPP1

**Fig. 5:**
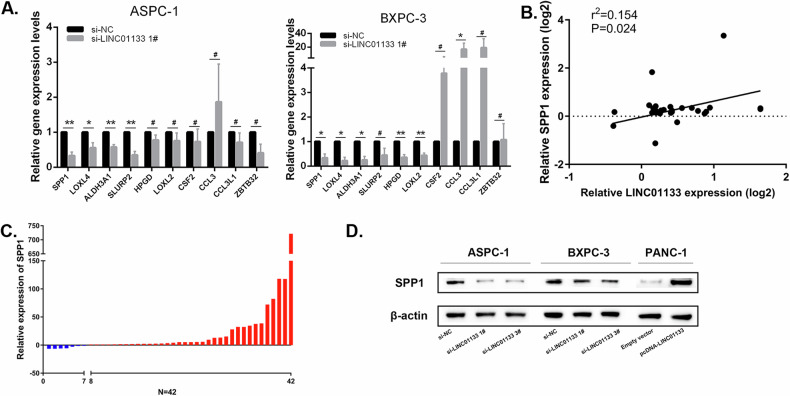
SPP1 was the downstream factor of LINC01133 and could promote tumor migration and EMT. **A** Relative expression of 10 differentially expressed genes selected by RNA sequencing in PDAC cell lines after LINC01133 knockdown. **B** Relative expression of SPP1 in PDAC tumor and adjacent tissue. **C** Relationship of SPP1 and LINC01133 expression in PDAC tumor by qRT-PCR. **D** SPP1 protein level measured by western blot after LINC01133 knockdown. Points, mean; bars, SD. *P < 0.05, **P < 0.01.

To validate the sequencing results, 10 genes with the highest log2 Fold Change score were submitted for qRT-PCR analysis in ASPC-1 and BXPC-3 cells transfected with s-LINC01133 1# and si-NC. SPP1, LOXL4, and ALDH3A1 expression significantly decreased in LINC01133 knockdown of both cell lines (Fig. 5A). SLURP2, HPGD, LOXL2, CSF2, CCL3, ZBTB32, and CCL3L1 expression was not significantly differed after LINC01133 knockdown in one or two cell lines. We found that SPP1 downregulation was most prominent after LINC01133 knockdown in both BXPC-3 and ASPC-1 cells, and submitted SPP1 for further analysis.

To determine whether LINC01133 can regulate SPP1 expression, quantitative measurement and function analysis were performed. The qRT-PCR confirmed that SPP1 expression was positively correlated with LINC01133 expression (Pearson correlation coefficient *r*^2^ = 0.154, *P* = 0.024, Fig. 5B). SPP1 was also significantly over-expressed in PDAC tissues compared to adjacent normal tissue (Fig. 5C). Protein level of SPP1 was decreased after LINC01133 knockdown (Fig. 5D). Taken together, we determined that SPP1 could be regulated by LINC01133 and was its downstream protein.

**Fig. 6:**
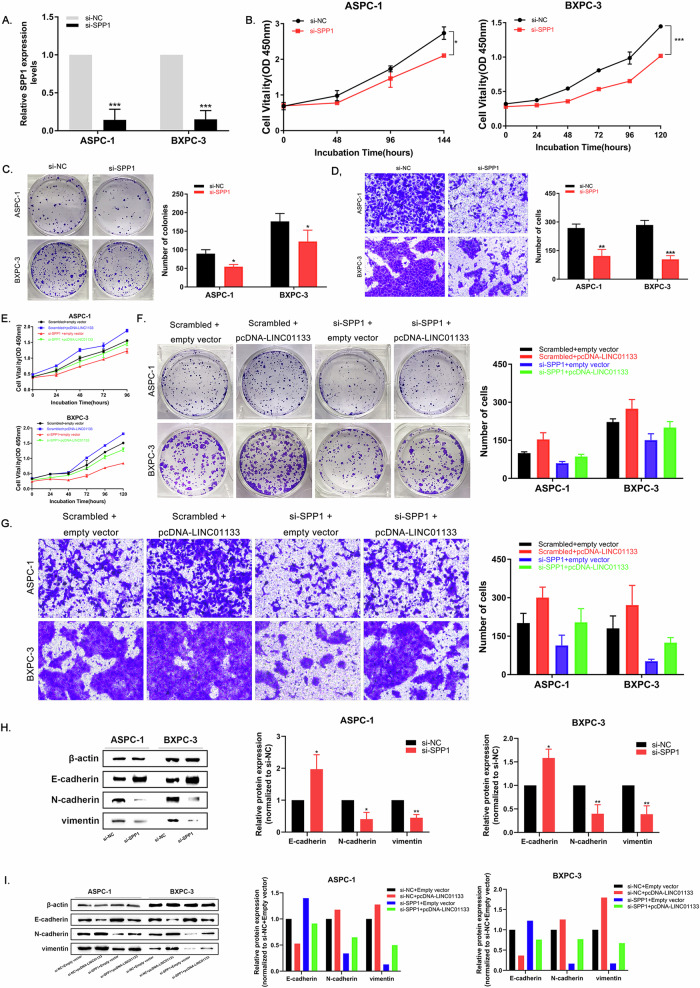
LINC01133 promoted tumor migration and EMT by targeting SPP1. **A** Si-SPP1 transfection could effectively knock down SPP1 expression in BXPC-3 and ASPC-1 cell lines. **B-C** Cell proliferation ability after SPP1 knockdown measured by CCK-8 and colony formation analysis. **D** Cell migration ability after SPP1 knockdown measured by transwell analysis. **E-F** Cell proliferation was measured by CCK-8 and colony formation analysis after co-transfected with si-SPP1 and pcDNA-LINC01133, with controls. **G** Cell migration was measured by transwell assay after co-transfected with si-SPP1 and pcDNA-LINC01133, with controls. **H** SPP1 protein level of BXPC-3 and ASPC-1 after transfected with si-SPP1 and negative control. **I** Western blot assay and relative protein level of EMT markers E-cadherin, N-cadherin, and vimentin in LINC01133 over-expressed and si-SPP1 groups, with controls.

To investigate the role of SPP1 in PDAC tumorigenesis, si-SPP1 was designed to knockdown SPP1 expression, which displayed an effective knockdown effect and was used for subsequent functional experiments (Fig. 6A). CCK-8 and colony formation analysis showed cell lines proliferation with down-regulated SPP1 was decreased (Fig. 6B, C). Cell migration was also inhibited after SPP1 knockdown as shown in transwell analysis (Fig. 6D). These results showed that SPP1 had a tumor-promoting ability as LINC01133. We co-transfected pcDNA-LINC01133 and si-SPP1 into ASPC-1 and BXPC-3 cells to see if SPP1 knockdown could reverse the tumor-promoting effect after LINC01133 over-expression. Down-regulation of SPP1 can partially reverse the pro-proliferation effect of over-expressing LINC01133 compared to cells transfected with negative control and pcDNA-LINC01133 (Fig. 6E–G). These results indicated that SPP1 functions as the downstream target of LINC01133 in PDAC.

Previous studies indicated that SPP1 was an important factor in tumor microenvironment formation [20]. So we investigated if SPP1 participated in the EMT process. Western blot analysis showed the expression of E-cadherin was increased, while N-cadherin and vimentin expression were decreased in the si-SPP1 group, suggesting SPP1 knockdown could suppress the EMT process in PDAC cells. (Fig. 6H). As we demonstrated before, LINC01133 over-expression promoted the EMT process in PDAC. We hypothesized that LINC01133-induced SPP1 over-expression may increase PDAC proliferation by promoting the EMT process. Knocking down SPP1 could partially reverse the pro-EMT effect after over-expressing LINC01133 (Fig. 6I), indicating that LINC01133 executed its EMT-promoting function via targeting SPP1.

### Arp3 bound with LINC01133 and promoted SPP1 expression

**Fig. 7:**
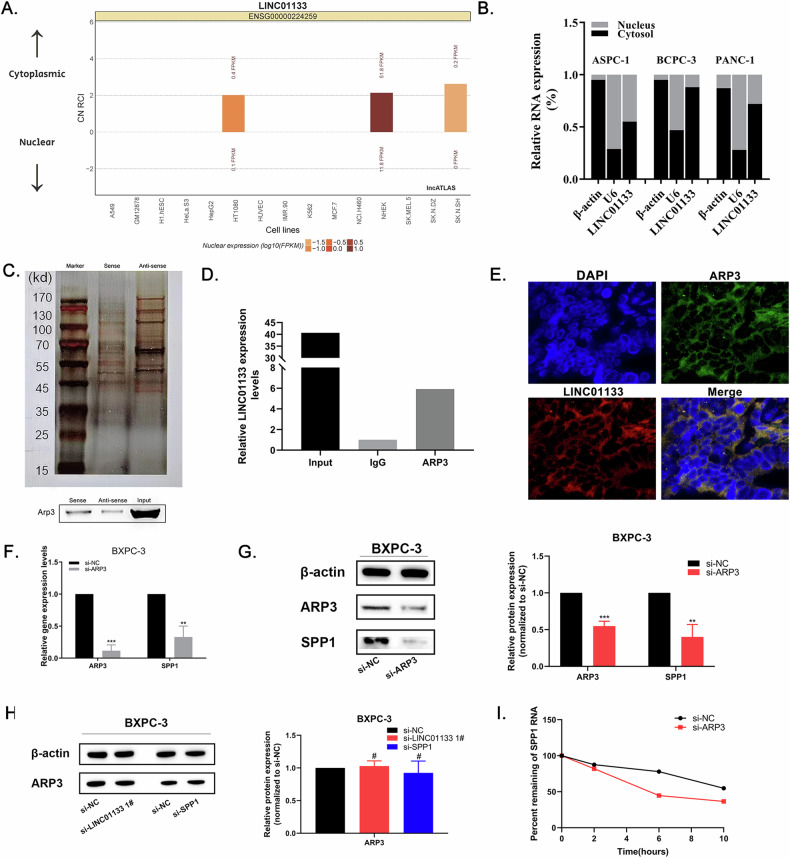
Arp3 bound with LINC01133 and promoted SPP1 expression. **A** LINC01133 was predicted located mainly in the cytoplasm by the online bioinformatic analysis website LncATLAS. **B** Subcellular fractionation location showed LINC01133 mainly located in the cytoplasm of 3 PDAC cell lines. **C** Western blot analysis after LINC01133 pull-down showed a higher Arp3 protein level in the sense LINC01133 pull-down product. **D** The qRT-PCR result of LINC01133 level after RIP analysis using anti-Arp3 antibody. **E** ARP3 and LINC01133 expression in PDAC tumor tissue by immunofluorescence and FISH. **F** Relative expression of ARP3 and SPP1 in PDAC cell line BXPC-3 after ARP3 knockdown. **G** ARP3 and SPP1 protein levels were measured by western blot after ARP3 knockdown. **H** ARP3 protein level measured by western blot after LINC01133 and SPP1 knockdowns. **I** The RNA degradation of SPP1 in ARP3 knockdown and control groups. RIP, RNA immunoprecipitation. FISH, fluorescence in situ hybridization.

LINC01133 was predicted located mainly in the cytoplasm using lncATLAS (Fig. 7A). Subcellular localization confirmed that LINC01133 was mainly located in the cytoplasm of PDAC cells (Fig. 7B). To determine the molecular mechanism of LINC01133 in PDAC, pull-down assay was conducted to explore the interacting protein of LINC01133. The Arp3 protein level was higher in the biotin-labeled LINC01133 sense pull-down product than in the anti-sense product, showing that Arp3 could directly bind with LINC01133 (Fig. 7C). We further verified the result using RIP assay, in which the LINC01133 was significantly enriched in Arp3 antibody treatment unit than in IgG control unit (Fig. 7D). IF-FISH showed that LINC01133 and Arp3 were colocalized in the cytoplasm of tumor cells in PDAC tissue microarray (Fig. 7E).

Both Arp3 and SPP1 were involved in extracellular matrix regulation [21, 22], but no research was done on their interaction mechanism. BXPC-3 cells were treated with si-ARP3, si-SPP1 or negative control, and knockdown of ARP3 led to lower expression of SPP1 on both RNA and protein levels in BXPC-3 cells, while SPP1 or LINC01133 knockdown did not affect ARP3 mRNA or protein expression as shown in qRT-PCR and western blot (Fig. 7F–H). This indicated that Arp3 was the upstream regulator of SPP1.

### LINC01133 enhanced ARP3 function which increased SPP1 mRNA stability and enhanced the PDAC EMT process

To investigate how Arp3 affects SPP1 expression, actinomycin D was administrated after ARP3 knockdown. After new RNA synthesis was blocked with actinomycin D, the loss of SPP1 was quantified by qRT-PCR assay. RNA degradation of SPP1 was significantly increased after ARP3 knockdown (Fig. 7I), suggesting that LINC01133 and ARP3 co-regulated the expression of SPP1 in PDAC by stabilizing SPP1 RNA. Since LINC01133 was mainly located in the cytoplasm of PDAC cells, we hypothesized that the LINC01133 may enhance the function of Arp3 after binding, leading to increased SPP1 RNA stability and eventually SPP1 overexpression.

### C-Jun activates LINC01133 transcription after β-catenin pathway activation

**Fig. 8:**
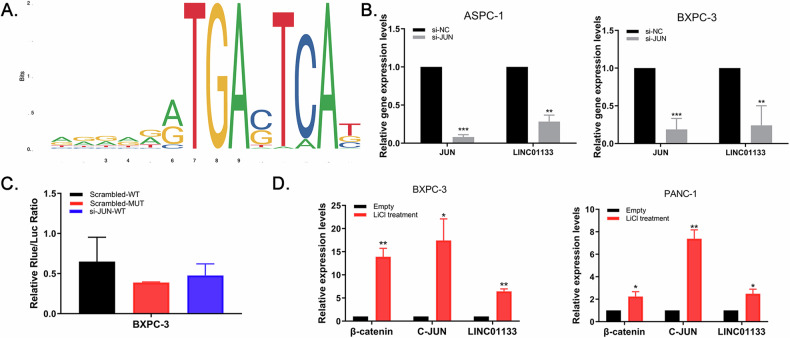
C-Jun bound to the promoter region of LINC01133 and activates its transcription. **A** Online websites predicted that transcription factor C-Jun could bind to the promoter of LINC01133. **B** LINC01133 RNA level after c-Jun knockdown showed a marked correlation between the two factors. **C** Luciferase activity in BXPC-3 cells after co-transfected si-JUN with LINC01133 promoter-mutated or promoter-wild-type vectors. **D** RNA expression of β-catenin, c-JUN, and LINC01133 after WNT/β-catenin pathway activation by LiCl treatment in BXPC-3 and PANC-1.

Online websites hTFtargetk [23], PROMO, and JASPAR [24] used to perform promoter analysis predicted that the LINC01133 promoter could bind to the transcription factor Jun proto-oncogene (c-Jun) (Fig. 8A). C-Jun siRNA was transfected into the BXPC-3 cells and qRT-PCR analysis revealed that LINC01133 expression correlated with c-Jun and was significantly decreased after c-JUN knockdown (Fig. 8B). Dual-luciferase reporter assay showed that relative luciferase activity after c-Jun knockdown was decreased compared to control (Fig. 8C). These findings suggested that c-JUN upregulated LINC01133 expression by binding to its promotor region and activating LINC0113 transcription.

Because c-Jun was a reported downstream molecule of β-catenin [25], we thought to investigate if the WNT/β-catenin pathway could affect LINC01133 in PDAC. After being treated with 20 mM/well WNT signaling agonist lithium chloride (LiCl), RNA expression of CTNNB1 (β-catenin), c-JUN, and LINC01133 in BXPC-3 and PANC-1 were all increased compared to controls (Fig. 8D), suggesting WNT/β-catenin pathway activation could promote LINC01133 expression.

## Discussion

In the present study, we constructed a novel PDAC genetic risk model and explored the function of one of the key factors, LINC01133, and its role in promoting the EMT process in PDAC. LINC01133 was upregulated and could promote tumor cell proliferation in PDAC. Pathway enrichment analysis, qRT-PCR, and subsequent western blot tests revealed LINC01133 expression positively correlated to SPP1, their over-expression led to enhanced EMT process in PDAC. Pull-down and RIP assays found that LINC01133 bound with Arp3, the complex reduced SPP1 mRNA degradation which increased SPP1 mRNA level, ultimately leading to PDAC proliferation.

Previously, LINC01133 has emerged as an important cancer-related gene and a growing number of studies have gradually uncovered its role in cancer. LINC01133 may execute its biological function in a tissue-specific manner as it has diverse expressions and effects on tumor progression in different cancers. Its role in the EMT process is also varied. In gastric cancer and colorectal cancer, LINC01133 acts as a tumor suppressor and is downregulated in tumor cells, inhibiting the EMT process [15, 17, 26]. LINC01133 is up-regulated in PDAC and is associated with PDAC tumor growth, proliferation, migration, metastasis, and invasion [27, 28]. Consistent with previous studies, our study found LINC01133 was upregulated in PDAC tissue and cellular level, and LINC01133 over-expression could promote PDAC proliferation and migration in vivo and in vitro.

The downstream tumor-promoting mechanism of LINC01133 was also explored by previous researchers. LINC01133 could sponge with microRNAs and promote PDAC development [29, 30]. Our RNA sequencing result suggested SPP1 expression correlated with LINC01133. SPP1 is a protein-coding gene that produces a multi-functional matricellular phosphoglycoprotein, also known as osteopontin. SPP1, or osteopontin, was found able to guide EMT through multiple cellular signaling pathways and by restructuring the tumor microenvironment to modify EMT processes [31]. SPP1 promotes fibrosis and is critically involved in the fibroblast to myofibroblast transformation, which participates in tumor proliferation, angiogenesis, and metastasis [32, 33]. It also generates cancer-associated fibroblasts from normal fibroblasts and mesenchymal stem cells, leading to increased TGF-β and IL-6 secretion and inducing EMT [32]. We were the first study to link SPP1 and EMT process with LINC01133 over-expression, demonstrating a novel SPP1 regulation network that may be beneficial in future anti-cancer therapy. Interestingly, another study on the function of exosomal LINC01133 found that LINC01133 could be regulated by periostin, which was another extracellular matrix protein secreted by pancreatic stellate cells, leading to PDAC EMT [18]. Taken together, these suggested that LINC01133 could interact with various extracellular components and play a key role in the EMT process in PDAC.

Previous studies have indicated that LINC01133 is located in both nuclear and cytoplasm and can execute its function in both locations [18, 29]. We found LINC01133 mainly located in the cytoplasm of PDAC cells. RIP analysis showed that LINC01133 could bind with Arp3 in PDAC. The RNA stability test also confirmed our hypothesis that Arp3 inhibited SPP1 degradation. Arp3, also known as actin-related protein 3, was a subunit of the Arp2/3 complex, the activation of which was reported associated with an early PDAC carcinogenesis process known as acinar-to-ductal metaplasia [34]. A subunit of the Arp2/3 complex could regulate cell migration and invasion in PDAC, and a decrease in cell migration ability could be observed after silencing the Arp2/3 complex [35, 36]. Various studies have elaborated on the direct role Arp2/3 played in the formation of branched actin network. Here, we demonstrated a novel Arp3 regulatory pathway in PDAC that could potentially be used as a therapeutic target.

Bioinformatic analysis inferred that c-Jun can bind to the promoter region of LINC01133 and promote LINC01133 transcription. Our experiments confirmed that c-Jun expression correlated with LINC01133. C-Jun is a transcription factor that is closely associated with tumorigenesis and tumor metastasis, and a downstream modulator of the WNT/β-catenin signaling pathway [37]. Previous studies have demonstrated a positive correlation between LINC01133 and c-Jun, further corroborating our result [38].

In summary, we developed a novel prognostic model of PDAC based on gene signature and investigated the oncogenic functions of a key gene, LINC01133. We found that LINC01133 could bind with Arp3 forming a complex that could increase SPP1 RNA stability, leading to enhanced EMT process in PDAC. The process could be regulated by WNT/β-catenin pathway activation through c-JUN over-expression. Together, we demonstrated a novel pathway that could induce EMT in PDAC and hopefully could improve the understanding of the interaction between lncRNA and cancer for potential diagnostic and therapeutic purposes.

### Supplementary information

Supplementary Materials and Methods

Supplemental legends

Supplementary figure S1

Supplementary table 1 and 2

Original western blot data

## Data Availability

All data generated or analyzed during this study are included in this published article and its supplementary information files. The RNA sequencing datasets generated in this study are deposited in the GEO database (GSE267702).
