# Supplementary material for: LINC01133 promotes pancreatic ductal adenocarcinoma epithelial–mesenchymal transition mediated by SPP1 through binding to Arp3
Source: Cell Death Dis. 2024 Jul 10;15(7):492. doi: 10.1038/s41419-024-06876-3 (PMC11237081; doi:10.1038/s41419-024-06876-3)
Supplement: Supplementary file 1 — Supplementary Materials and Methods [file 41419_2024_6876_MOESM1_ESM.docx]

**Supplementary Materials and Methods**

**Cell culture**

Human PDAC cell lines BXPC-3, ASPC-1, PANC-1, CFPAC-1, and Mia CaPa-2 as well as normal human pancreatic epithelial HPNE were purchased from Zhong Qiao Xin Zhou Biotechnology Co., Ltd (Shanghai, China) and cultured in RPMI 1640 medium (Gibco Life Technologies, Grand Island, NY, USA) or DMEM (Gibco Life Technologies) according to supplier’s instructions. All cell lines have been authenticated. The medium was added with 10% fetal bovine serum (AusGeneX, Brisbane, Australia) and 1% penicillin and streptomycin (Invitrogen Co., Ltd, Carlsbad, CA, USA). Cells were incubated under a 5% CO2 humidified atmosphere at 37°C.

**Cell transfection**

Three LINC01133 small interfering RNAs (si-RNAs, si-LINC01133 1#/2#/3#), one SPP1 siRNA, one ARP3 siRNA, one C-JUN siRNA and scrambled negative control siRNA (si-NC) designed and purchased from Invitrogen were transfected to PDAC cells together with Lipofectamine 3 000 (Invitrogen Co., Ltd) according to the manufacturer’s instructions. The Full-length complementary cDNA of LINC01133 was synthesized and cloned into the expression vector pcDNA3.1 (Genebay, Nanjing, China), and transfected into PDAC cells by RFect Plasmid DNA Transfection Reagent (Proteinbio, Nanjing, China) following the manufacturer’s protocol to create LINC01133 over-expression cells (pcDNA-LINC01133). The transfected cells were collected 48 hours after transfection for further analysis.

**CCK-8 test**

Cell Counting Kit-8 (CCK-8; Bimake, Houston, TX, USA) was used to measure cell proliferation. Transfected PDAC cells were seeded onto 96-well plates with a density of 6 000 cells/well. 20 μl CCK-8 reagent was added to each well and then cultured for 2 h to measure cell viability every 24 h. All tests were replicated 3 times.

**Colony formation test**

Transfected cells were seeded in 6-well plates and cultured for 14 days for colony formation assay. Methanol and 0.1% crystal violet were used to fix and stain the cells in each well. Visible colonies were counted.

**Transwell analysis**

Transfected PDAC cells were added into the Transwell chamber (8 µm pore diameter; Corning Incorporated, Corning, NY, USA) with a density of 15 000 cells/chamber to test cell migration. Cells that passed through the membrane after 48 h were counted under the microscope after being fixed by methanol and stained with crystal violet.

**Ethynyldeoxyuridine (EdU) analysis**

Cells were seeded into 24-well plates and transfected. EdU cell proliferation kit (Ribobio, Guangzhou, China) was used to measure cell proliferation according to the manufacturer’s protocol. 50 μM EdU labeling medium was added into wells 48h after transfect and incubated for 2 hours. The mixture was fixed with 4% paraformaldehyde for 30 min and then treated with 0.5% Triton X-100 for 20 min at room temperature. Cells were then stained with DAPI staining solution. Five fields of view under the fluorescent microscope were captured to calculate the percentage of EdU-positive cells.

**Flow cytometry analysis**

BXPC-3, ASPC-1 and PANC-1 cells transfected with si-LINC01133 or si-NC were harvested 48h after transfection for flow cytometry analysis. For apoptosis analysis, cells were stained with FITC Annexin V and propidium iodide (PI) using the FITC Annexin V Apoptosis Detection Kit (BD Biosciences, San Jose, CA, USA) following the manufacturer’s protocol. The cells were classiﬁed into viable, dead, early apoptotic, and apoptotic cells, and then the ratio of early apoptotic cells was compared with the control. For cell cycle analysis, the Cell Cycle Assay Kit (including RNase A) (FMS-CCC01, FcMACS, Nanjing, China) was used following the manufacturer’s protocol. The percentage of cells in the G0/G1, S, and G2/M phases was calculated. The cells were analyzed by ﬂow cytometry (FACScan; BDBiosciences).

**Subcellular fractionation location**

The separation of nuclear and cytosolic fractions was performed using the Cytoplasmic & Nuclear RNA Purification Kit (Norgen Biotek, Thorold, Canada) according to the manufacturer’s instructions. The qRT-PCR test was performed for further quantitive evaluation.

**Tumor formation and tail vein metastasis nude mice model construction**

The animal study was approved by the Animal Ethical and Welfare Committee of Nanjing Medical University (IACUC-1601248). 4-week-old female BALB/c nude mice purchased from Charles River (Beijing, China) were kept under specific pathogen-free conditions in the animal center of Nanjing Medical University.

BXPC cells were transfected with si-NC or si-LINC01133 1# RNA as mentioned above, and cells were harvested and suspended at a concentration of 1 × 10^8^ cells/ml and kept on ice before injection. 5 mice were injected with 100 μl suspended cells with different treatments on either side of the armpit. Every 6 days, tumor size was measured and mice were euthanized by inhalation of high concentrations of carbon dioxide before the tumor reached 1.5 cm in the largest diameter and the subcutaneous tumors harvested were measured and weighed.

10 mice were randomly divided into 2 groups and their tail veins were injected with a total of 100 μl suspended BXPC cells transfected with si-NC or si-LINC01133 1# RNA at a concentration of 1 × 10^6^ cells/ml. 28 days after injection, the nude mice were euthanized and their organs were removed to observe metastasis PDAC tumor nodules. Organs were resected every 2 mm and the entire lung and liver were submitted for pathological evaluation. H & E staining and immunohistochemical staining of Ki-67(Maxim Biotechnology Development Co. LTD, Fuzhou, China) was performed on harvested samples and the Ki-67 index was interpreted by two pathologists using a double-blinded method, and a third pathologist was invited for review if their results have an over 10% difference.

**Transcriptome high-throughput sequencing**

BXPC-1 cells were planted in a six-well plate and lysed with 1 ml TRIzol per well 48 h after transfection with si-LINC01133 1#. 200ul of lysate was used and submitted for qRT-PCR analysis to confirm interference efﬁciency and the other 800ul lysate was sent for high-throughput sequencing by BGI (Wuhan, China). Gene Ontology (GO), Kyoto Encyclopedia of Genes and Genomes (KEGG), and Gene Set Enrichment Analysis (GSEA) were used to conduct enrichment analysis to analyze the genes under LINC01133 regulation.

**Immunofluorescence and fluorescence *in situ* hybridization (IF - FISH)**

Tissue microarray was prepared using formalin-fixed paraffin-embedded (FFPE) tumor tissue blocks from the 40 patients mentioned above, with a diameter of 1.5 mm for each sample. Slides that underwent antigen retrieval were permeabilized with 0.5% Triton X-100 for 5 min and dehydrated before the RNA probe of LINC01133 (Ribobio, Guangzhou, China) was added to separate slides and hybridized overnight at 37˚C. Slides were then rinsed and were blocked for 30 min before being incubated with primary antibodies (1: 200) of SPP1 and Arp3 (Proteintech) overnight at 4°C and then with the corresponding second antibody for 1 h at room temperature. Nuclei were stained with DAPI. Cellular fluorescence was observed via an Olympus BX53 microscope.

**Dual-luciferase reporter assay**

The potential binding site of c-Jun on LINC01133 promoter region was analyzed and promoter-mutated and promoter-wild-type vectors were constructed and transfected into BXPC-3 cells plated in 96-well plates. The relative activity of luciferase was tested according to the protocol of Dualucif® Firefly & Renilla Assay Kit (UElandy, Suzhou, China).

**RNA pull-down**

Full-length sense and antisense LINC01133 sequences were obtained using Thermo Scientific Pierce RNA 3’Desthiobiotinylation Kit (Thermo Fisher Scientific, Waltham, MA, USA). RNA pull-down assays were performed using Pierce^TM^ Magnetic RNA-Protein Pull-down Kit (Thermo Fisher Scientific) according to the manufacturer’s protocols. The product was subjected to western blot assay for protein analysis.

**RNA** **immunoprecipitation (RIP) assay**

RIP was performed using the EZ-Magna RIP^TM^ RNA-Binding Protein Immunoprecipitation Kit (Millipore) following the manufacturer’s protocol. BXPC-3 cells were scraped off and extracted in a complete RIP lysis buffer. A total of 100 μl of whole cell extract was incubated with RIP buffer containing magnetic bead-antibody complex against Arp3 or control IgG (Millipore) overnight at 4 °C with rotating. The beads were then washed with washing buffer, the complexes were incubated with 0.1% SDS/0.5 mg/ml Proteinase K (30 min at 55 °C) to remove proteins. The immunoprecipitated RNA was puriﬁed and analyzed by qRT-PCR.

**RNA stability test**

Cells were planted into 6-well plates and subjected to transfection. Actinomycin D (MedChemExpress, Monmouth Junction, NJ, USA) was added into medium 2 days after transfection to block RNA transcription in a concentration of 1 μg/mL for 0, 2, 6, and 10 h, and then RNA was extracted for RT-qPCR detection.
