# Supplementary material for: LINC01133 promotes pancreatic ductal adenocarcinoma epithelial–mesenchymal transition mediated by SPP1 through binding to Arp3
Source: Cell Death Dis. 2024 Jul 10;15(7):492. doi: 10.1038/s41419-024-06876-3 (PMC11237081; doi:10.1038/s41419-024-06876-3)
Supplement: Supplementary file 2 — Supplemental legends [file 41419_2024_6876_MOESM2_ESM.docx]

**Supplemental figure and table legends**

**Supplemental figure S1** Cell cycle by flow cytometry analysis after LINC01133 knockdown in ASPC-1 and BXPC-3 cell lines, and LINC01133 over-expression in PANC-1 cell line.

**Supplementary Table S1** Primers’ sequences.

**Supplementary Table S2** Clinicopathological features between high and low risk groups.
