# Supplementary figures and images for: LINC01133 promotes pancreatic ductal adenocarcinoma epithelial–mesenchymal transition mediated by SPP1 through binding to Arp3
Source: Cell Death Dis. 2024 Jul 10;15(7):492. doi: 10.1038/s41419-024-06876-3 (PMC11237081; doi:10.1038/s41419-024-06876-3)

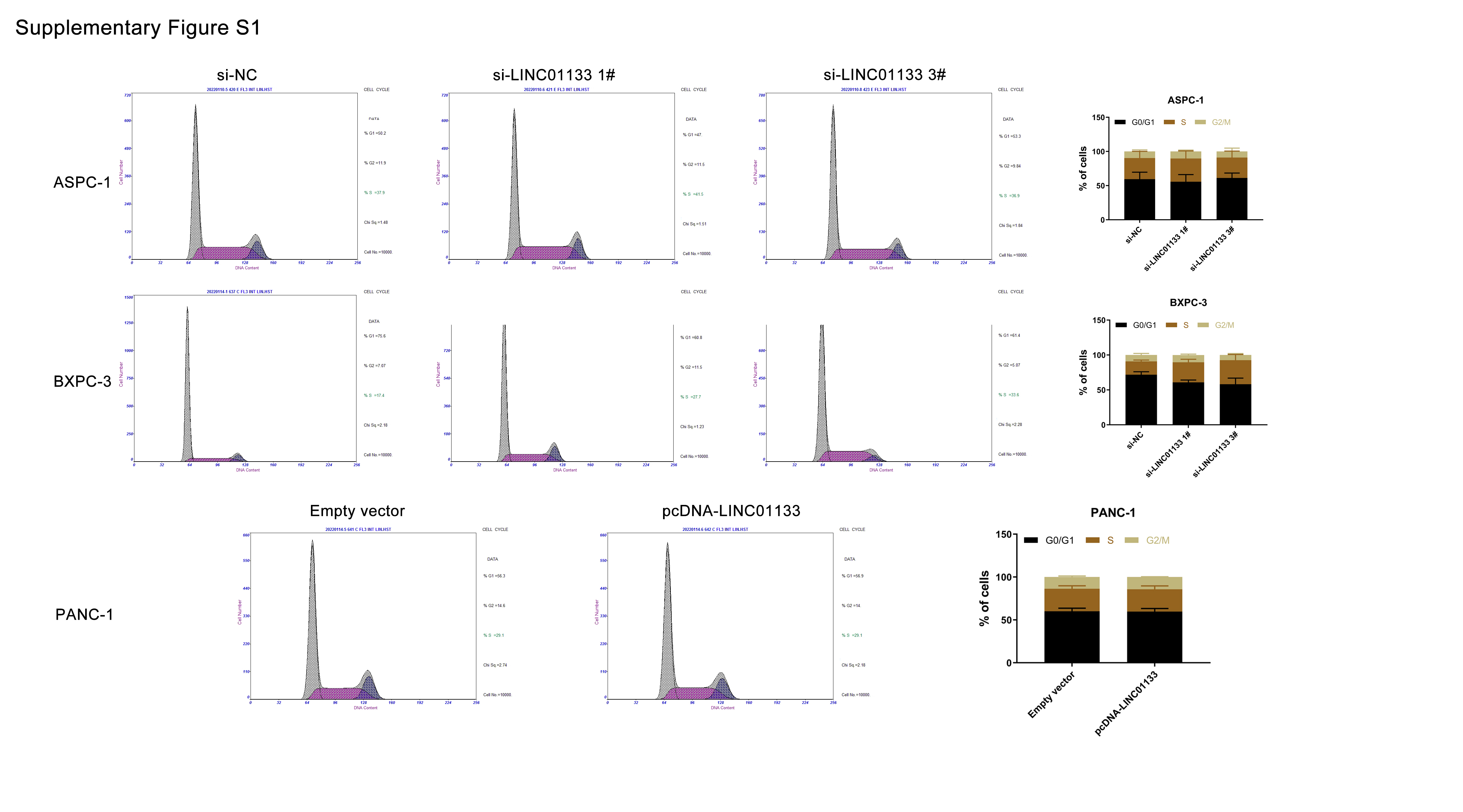

Supplement: Supplementary file 3 — Supplementary figure S1 [file 41419_2024_6876_MOESM3_ESM.jpg]

Figure 4D

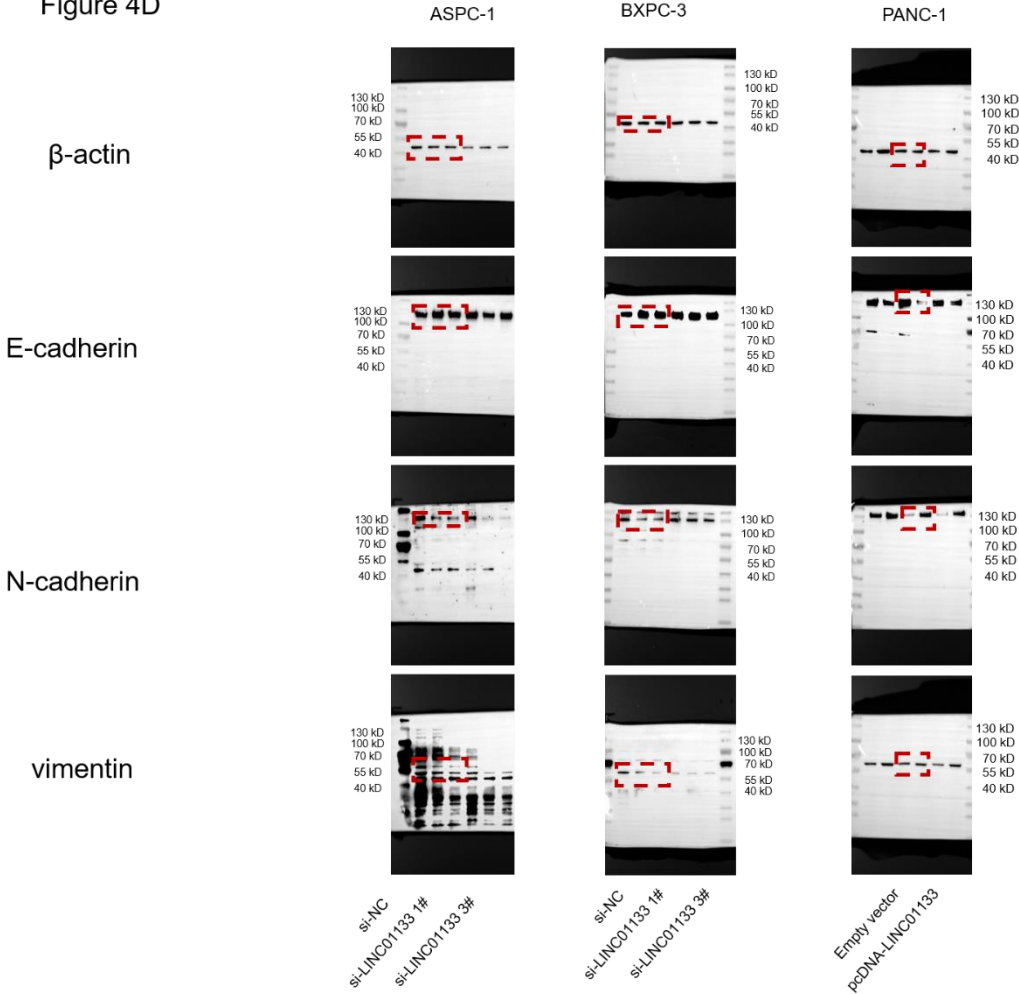

Figure 5D

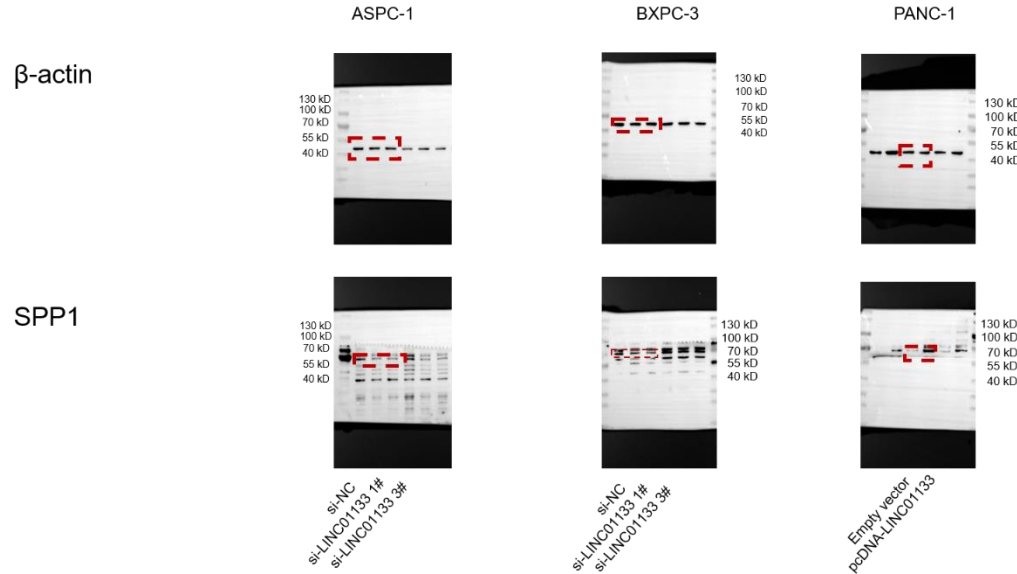

Figure 6H

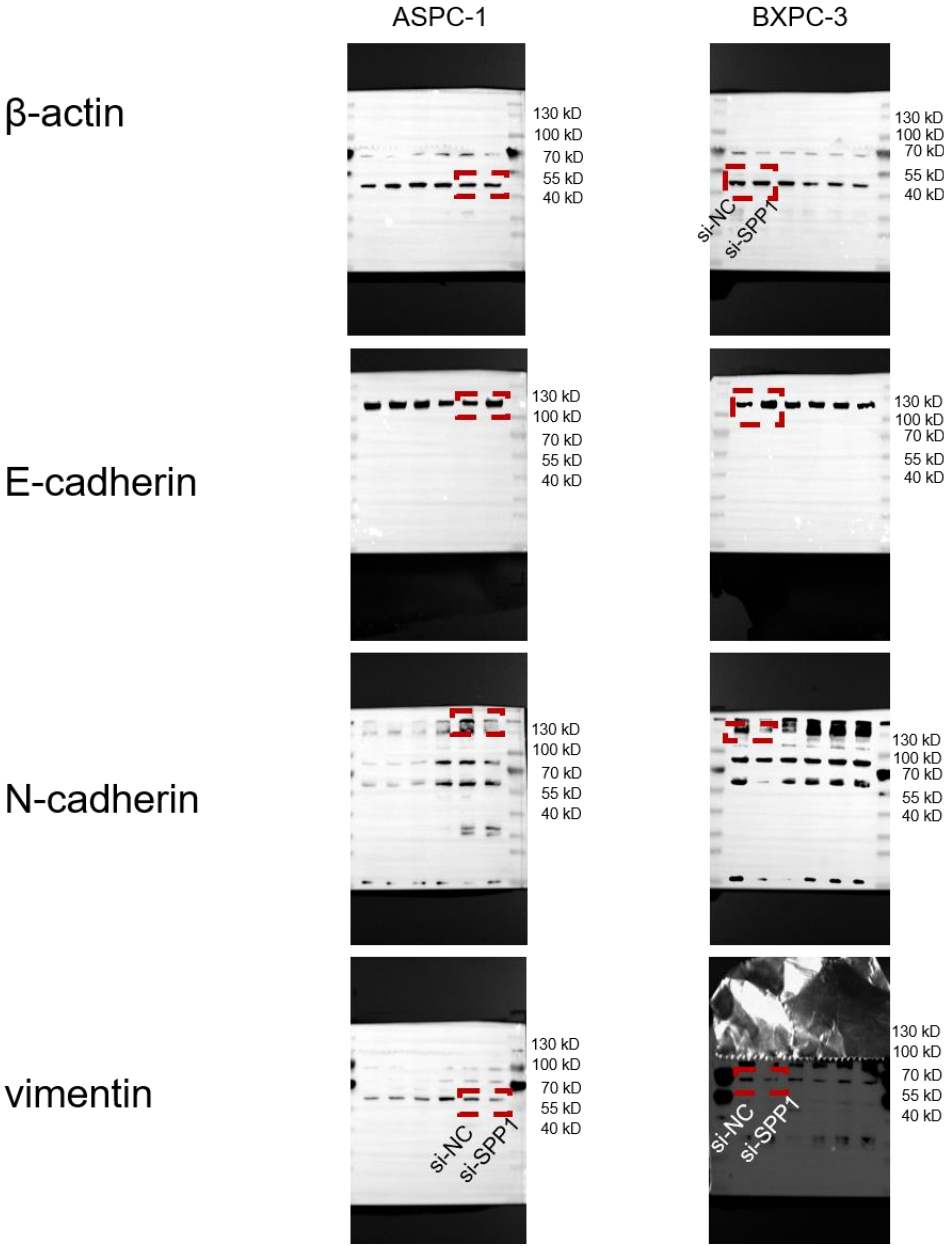

Figure 6I

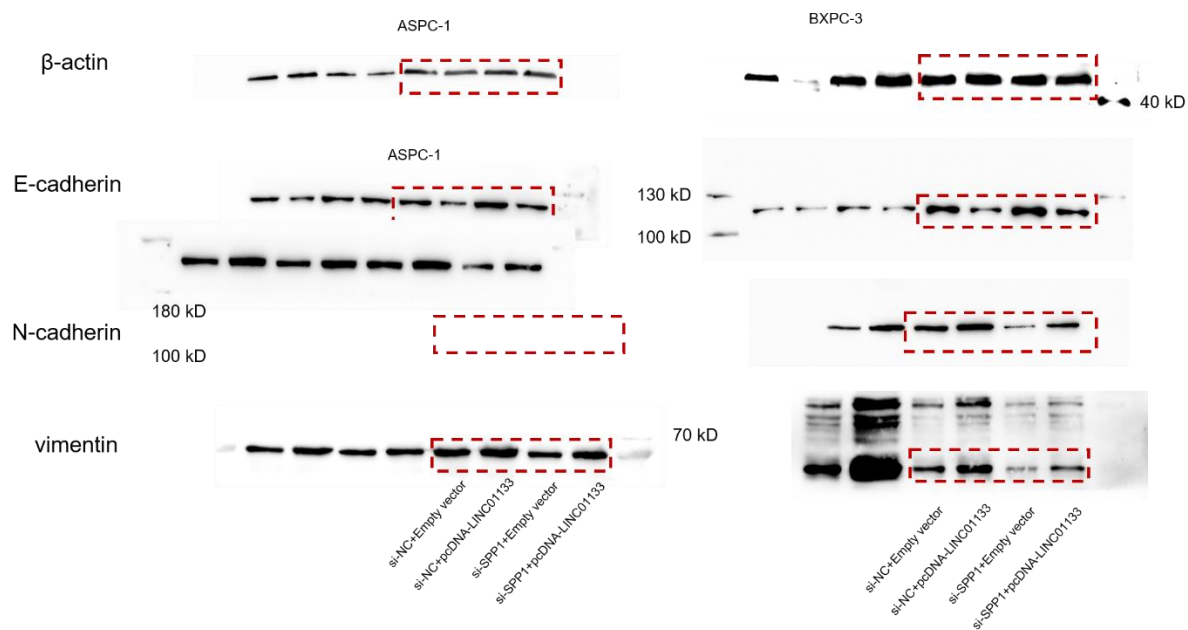

Figure 7C

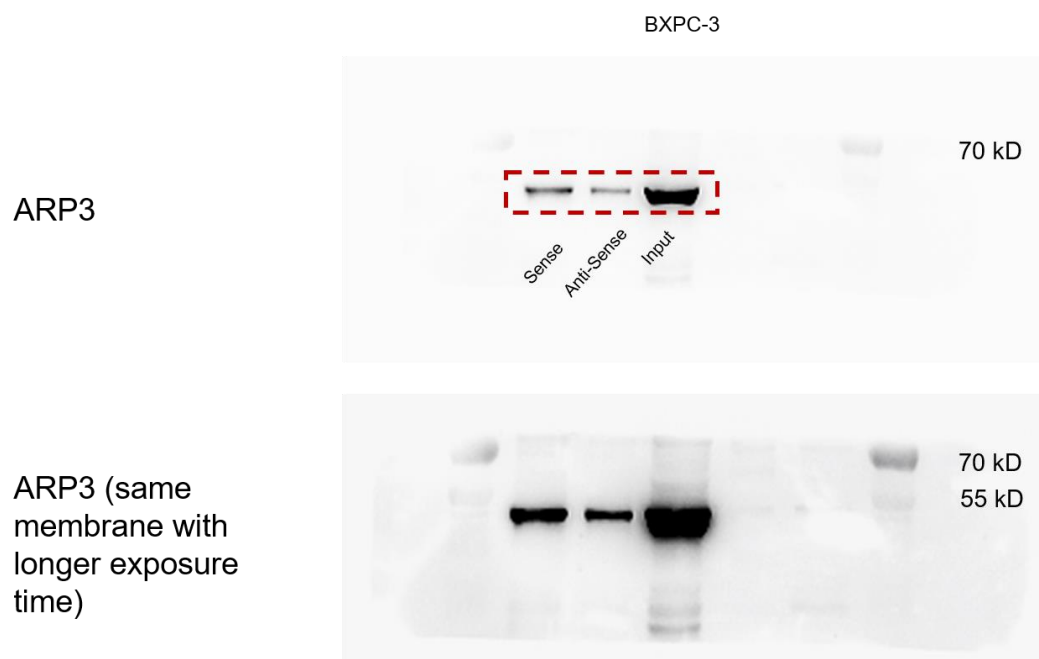

Figure 7F

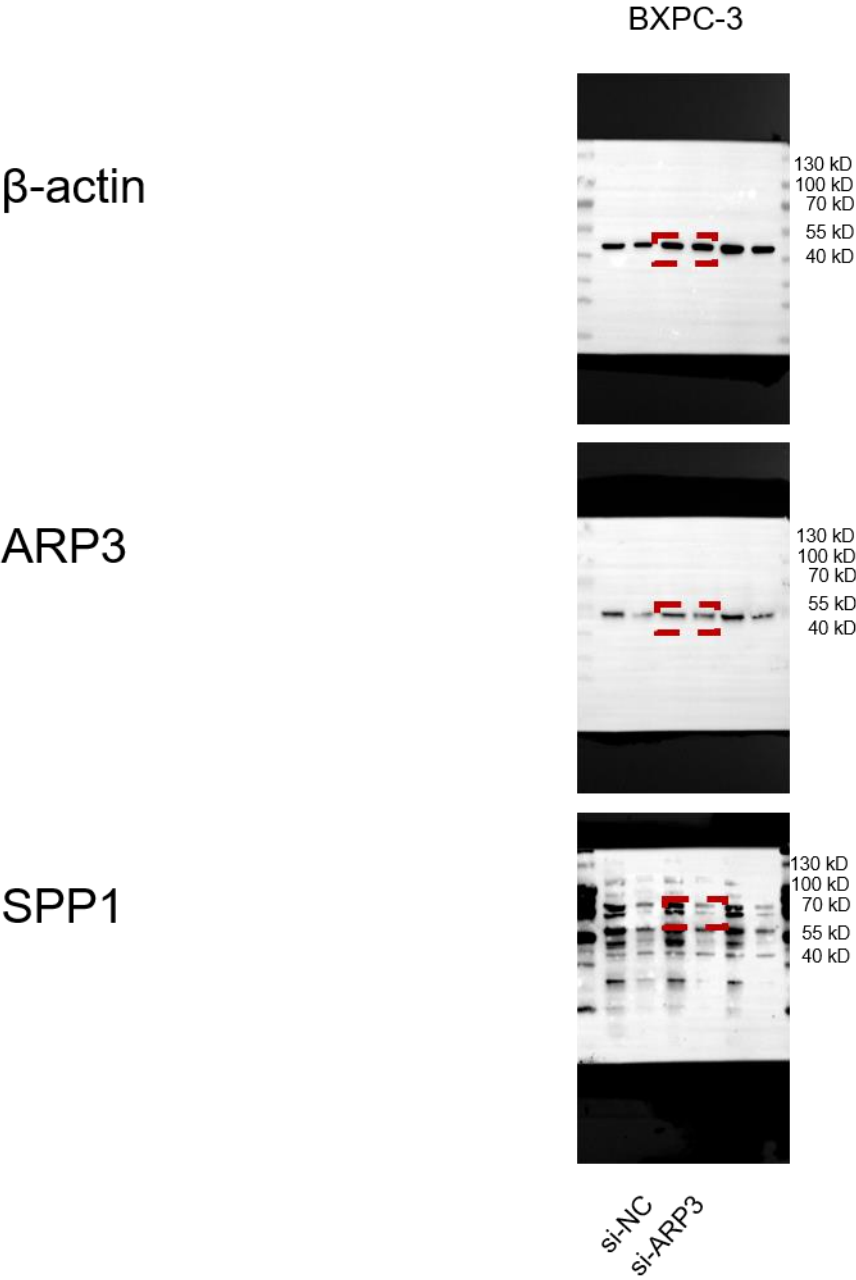

Figure 7G

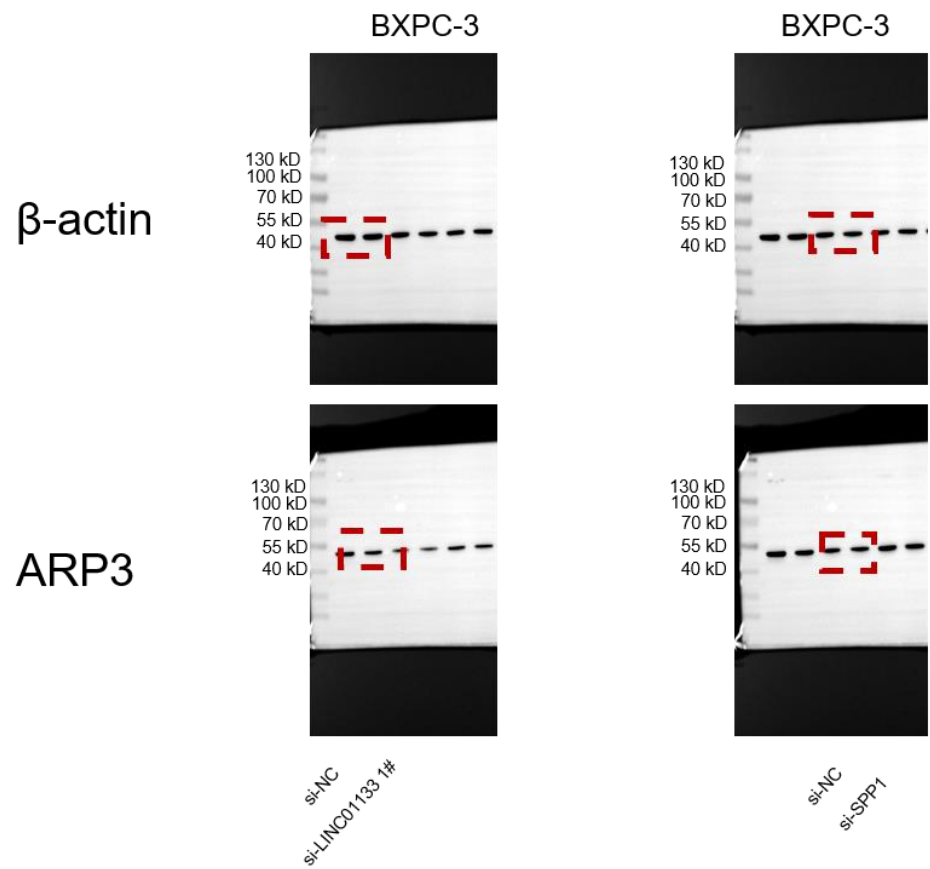

Supplement: Supplementary file 5 — Original western blot data [file 41419_2024_6876_MOESM5_ESM.pdf]
