# Supplementary material for: LINC01133 promotes pancreatic ductal adenocarcinoma epithelial–mesenchymal transition mediated by SPP1 through binding to Arp3
Source: Cell Death Dis. 2024 Jul 10;15(7):492. doi: 10.1038/s41419-024-06876-3 (PMC11237081; doi:10.1038/s41419-024-06876-3)
Supplement: Supplementary file 4 — Supplementary table 1 and 2 [file 41419_2024_6876_MOESM4_ESM.docx]

Table S1 Primers’ sequences

| Gene | Sequence |
| --- | --- |
| LINC01133, | (forward) 5’- AGGATGGATTCTCCCATTCC -3’ |
|  | (reverse) 5’- ATGAAACAATGCCAACAGCA -3 |
| SPP1 | (forward) 5’- CGCAGACCTGACATCCAGTA -3’ |
|  | (reverse) 5’- TGCTCATTGCTCTCATCATTG -3’ |
| ARP3 | (forward) 5’- CCTGTGGCTGAAGGGTATGT -3’ |
|  | (reverse) 5’- TCCTACTTCTCGGTCTCTCAGC -3’ |
| c-Jun | (forward) 5’- GAGCGGACCTTATGGCTACA -3’ |
|  | (reverse) 5’- CCGTTGCTGGACTGGATTAT -3’ |

Table S2 Clinicopathological features between high and low risk groups

|  | level | High-risk | Low-risk | P value |
| --- | --- | --- | --- | --- |
| n |  | 86 | 85 |  |
| age (%) | <65 | 40 (46.51) | 50 (58.82) | 0.1446 |
|  | ≥65 | 46 (53.49) | 35 (41.18) |  |
| sex (%) | male | 50 (58.14) | 43 (50.59) | 0.4022 |
|  | female | 36 (41.86) | 42 (49.41) |  |
| stage (%) | I-II | 82 (95.35) | 79 (96.34) | 1 |
|  | III-IV | 4 (4.65) | 3 (3.66) |  |
| ELN (%) | <12 | 32 (38.10) | 24 (28.92) | 0.2746 |
|  | ≥12 | 52 (61.90) | 59 (71.08) |  |
| grade (%) | G1 | 8 (9.41) | 20 (23.81) | 0.0261 |
|  | G2 | 49 (57.65) | 43 (51.19) |  |
|  | G3 | 28 (32.94) | 19 (22.62) |  |
|  | G4 | 0 (0.00) | 2 (2.38) |  |
| PLN (%) | 0 | 20 (23.81) | 26 (31.33) | 0.4252 |
|  | 1-3 | 39 (46.43) | 31 (37.35) |  |
|  | ≥4 | 25 (29.76) | 26 (31.33) |  |
